# Functional Role of Internal and External Visual Imagery: Preliminary Evidences from Pilates

**DOI:** 10.1155/2018/7235872

**Published:** 2018-04-15

**Authors:** Simone Montuori, Giuseppe Curcio, Pierpaolo Sorrentino, Lidia Belloni, Giuseppe Sorrentino, Francesca Foti, Laura Mandolesi

**Affiliations:** ^1^Department of Movement Sciences and Wellbeing, University “Parthenope”, Via Medina 40, 80133 Naples, Italy; ^2^Department of Biotechnological and Applied Clinical Sciences, University of L'Aquila, Via Vetoio (Coppito 2), 67100 L'Aquila, Italy; ^3^Department of Engineering, University “Parthenope”, Centro Direzionale, Isola C4, 80143 Naples, Italy; ^4^Istituto di Diagnosi e Cura Hermitage Capodimonte, Via Cupa delle Tozzole 2, 80131 Naples, Italy; ^5^Institute of Applied Sciences and Intelligent Systems, CNR, Via Campi Flegrei 34, 80078 Pozzuoli, Naples, Italy; ^6^Department of Medical and Surgical Sciences, “Magna Graecia” University of Catanzaro, Viale Europa, 88100 Catanzaro, Italy; ^7^IRCCS Fondazione Santa Lucia, Via del Fosso di Fiorano 64, 00143 Rome, Italy

## Abstract

The present study investigates whether a functional difference between the visualization of a sequence of movements in the perspective of the first- (*internal* VMI-I) or third- (*external* VMI-E) person exists, which might be relevant to promote learning. By using a mental chronometry experimental paradigm, we have compared the time or execution, imagination in the VMI-I perspective, and imagination in the VMI-E perspective of two kinds of Pilates exercises. The analysis was carried out in individuals with different levels of competence (expert, novice, and no-practice individuals). Our results showed that in the Expert group, in the VMI-I perspective, the imagination time was similar to the execution time, while in the VMI-E perspective, the imagination time was significantly lower than the execution time. An opposite pattern was found in the Novice group, in which the time of imagination was similar to that of execution only in the VMI-E perspective, while in the VMI-I perspective, the time of imagination was significantly lower than the time of execution. In the control group, the times of both modalities of imagination were significantly lower than the execution time for each exercise. The present data suggest that, while the VMI-I serves to train an already internalised gesture, the VMI-E perspective could be useful to learn, and then improve, the recently acquired sequence of movements. Moreover, visual imagery is not useful for individuals that lack a specific motor experience. The present data offer new insights in the application of mental training techniques, especially in field of sports. However, further investigations are needed to better understand the functional role of internal and external visual imagery.

## 1. Introduction

Sport psychology has shown that in order to achieve a favorable outcome in sport, it is necessary to integrate physical and mental practice [1, 2]. In this line of thinking, motor imagery (MI) has been studied extensively since it represents a potentially effective mean to promote learning [3, 4]. MI is defined as the mental execution of a movement, motor act, or action, without any overt movement or muscle activation [5–8] and represents a cognitive tool strategically used by athletes for optimizing their specific motor skills [9]. To underline how the two processes, physical and mental, are related, several studies showed that changes in physiological parameters, such as vegetative indices, are similar during MI and during execution [10–12]. Furthermore, neuroimaging data have demonstrated that the imagined and actual movements are functionally equivalent in the sense they share the same neural circuitry [13–15]. It could be hypothesized that such neural circuitry might underlie the improvements in movement efficiency induced by MI, allowing athletes to improve the level of performance [16, 17].

A modality of MI is represented by visuomotor imagery (VMI) that involves the visualization of a movement or a sequence of movements from the first- (*internal* VMI-I) or third- (*external* VMI-E) person perspective [18]. In the VMI-I perspective, the subjects imagine themselves from the same viewpoint experienced in the encoding phase [19, 20]. This perspective is greatly affected by the kinaesthetic features of the gesture and refers to the centredness of one's own multimodal experiential space upon one's own body, thus operating in an egocentric reference frame [21]. Conversely, the VMI-E perspective requires individuals to imagine themselves or others from an external point of view, observing the gesture as an onlooker [3, 19, 22]. In this modality, the subjects imagine the environment as well, the “background” of the scene, thus operating in an allocentric reference frame. The VMI-E perspective is considered to be a more complex mental process than the VMI-I perspective, because allocentric operations have to be integrated within the egocentric coordinates of the subject that is imagining, hence requiring an additional mental process [18].

In the last years, a much debated issue in sport psychology is whether internal or external VMI would be more efficient for athletes. Despite the multitude of studies present in the literature, this issue has not been investigated systematically. Namely, most of the research has focused either on specific types of sports, that is, open skill sport (such as karate, in which the participants fight in an environment that is changing rapidly) versus closed skill sport (such as gymnastics, in which the athletes perform in a relatively static environment) [23, 24], or on the level of competence of the athletes (i.e., experts versus novices) [25, 26]. Moreover, in the previous literature, the choice of the imagination perspective was based upon the athlete's preference (e.g., [27]).

From all these studies, several conflicting evidences can be drawn. With regard to the comparison between open skill sports and closed skill sports, it is shown that the VMI-E perspective is more used than VMI-I in open skill sports [18], while closed skill sport benefits from the VMI-I perspective [28, 29]. Such results are irrespective of the level of competence of the athletes. Moreover, the athletes specialized in open skill sport possess a high ability for mental processes and a better performance in the VMI-E perspective as compared to closed sport athletes [18]. These data could be explained by the notion that in open skill sports, the athletes move in environments that are rapidly changing and the VMI-E perspective includes such environments (e.g., opponents moving around). Other studies, based on the athlete's imagery preference, counteract these evidences. For example, it has been reported that athletes specialized in closed skill sport (e.g., golf) employed more imagery than those who participate in open sports (e.g., tennis and basketball) [30]. For what concerns the competence levels of the athletes, although imagery processes significantly help both novice and experienced athletes, the effects appear to be more pronounced for elite athletes [22, 25, 26], as demonstrated in studies with Olympic athletes [31–33]. To this regard, it has been highlighted that elite athletes, as compared to amateurs, had higher levels of imagery ability [27, 34, 35]. These data could be explained by the fact that elite athletes have had more opportunities to put into action imagery practice than lower level athletes [36, 37].

In the present study, we focused on the functional differences between internal and external perspectives, covering the issue from a different point of view. We set out to verify whether the two types of imagination improve different, yet integrated, cognitive-motor abilities in a closed skill discipline. This choice allows studying the VMI-E perspective in the contest of closed skill activities, differently from previous studies. Such an approach was chosen in order to study both perspectives separately, regardless of the specific kind of sport (closed or open skill).

In particular, we hypothesize that, while the VMI-I perspective revises a sequence of movements already internalised, the VMI-E perspective should be useful to learn, and improve, a recently acquired sequence of movements.

To address this issue, we used a mental chronometry paradigm in order to compare the time of execution of two Pilates exercises with the time of imagination of the same exercises in both VMI-I and VMI-E perspectives. Such experiment was carried out in individuals with different levels of competence (experts versus novices). As a control group, we analysed the execution and imagination time in individuals who had never practiced nor seen Pilates before.

We have chosen Pilates because it is a closed skill discipline, very much based on kinaesthetic features and practiced in stable and predictable conditions. Moreover, unlike other studies, we did not ask the participants to choose the modality of imagination they prefer, but we let them use both perspectives.

## 2. Materials and Methods

### 2.1. Participants

Forty-eight women (32 practicing Pilates, 16 not practicing Pilates) aged 24–58 years (mean age: 38.75 ± 11.25) participated in the study. According to their level of experience in the practice of Pilates, the participants were divided into 3 groups, each consisting of 16 women: *Expert group* (mean age: 39.25 ± 10.23), women who practiced Pilates regularly twice a week for the last twelve months at least; *Novice group* (mean age: 39.25 ± 12.82), women who started practicing Pilates no more than two months before the study; *no-Practice group* (mean age: 37.75 ± 11.2), women who have never practiced Pilates. We assessed through an informative questionnaire that women belonging to the *no-Practice group* had never seen a Pilates lesson or video or books showing Pilates. It is important to underline that, in order to perform Pilates correctly, it is essential to be aware of many aspects (motor control, breathing, etc.; see next paragraph) and that the knowledge of the sequence of movements to be performed is not enough. For this reason, the ones who have never practiced Pilates could not perform it correctly solely by imitation.

All participants did not have cardiovascular, neurological, or orthopaedic disorders and gave their written informed consent. They were recruited in sporting centres located in South Italy.

The study was conducted according to the 1964 Declaration of Helsinki.

### 2.2. Pilates Method and Exercises Chosen

Before considering the chosen exercises, it is appropriate to explain what Pilates is, designed by Joseph Pilates (1883–1967). Pilates is a type of discipline that pays great attention to self-awareness and to the pursuit of both physical and mental balance. It can be considered as a closed skill activity because the subjects perform the movements under a static and predictable environment. In particular, Pilates aimed at getting to know one's body and achieve its full acceptance. Through the improvement of concentration, breathing, balance, control, precision, and fluidity of movement, the individuals earn greater awareness of themselves, of their body, and of every single motor gesture.

**Figure 1:**
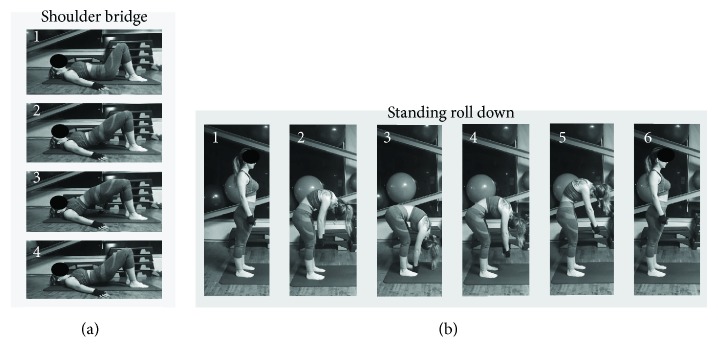
Sequences of the two Pilates exercises. (a) The shoulder bridge (SB) exercise: the individual lifts the pelvis whilst controlling breathing. Phase 1: start from the decubitus-supine position (1). Phase 2: the subject exhales. Then, the entire spine (vertebra by vertebra) is rolled up, until the weight is placed on the shoulder blades (2). Phase 3: the individual breathes in and holds still (3). Phase 4: while exhaling, the subject unrolls the spine (vertebra by vertebra) back to the starting position (4). (b) The standing roll down (SRD) exercise: the individual rolls down the spine, vertebra by vertebra, while controlling breathing. Phase 1: standing position with both legs extended at hip distance (1). Phase 2: the subject exhales and, starting from the cervical spine, rolls the whole spine (vertebra by vertebra), until the nose is at navel level (2). Phase 3: the individual inhales, relaxes, and abduces the shoulder blades, then exhales and moves the pelvis slightly forward, continuing to roll the vertebrae until the fingers touch the ground (3). Phase 4: the individual exhales and starts to unroll the spine vertebra by vertebra, beginning from the lumbar spine (4). Phase 5: the individual continues to unroll the spine, concentrating on the dorsal spine, keeping the arms relaxed (5). Phase 6: the individual returns to the starting position (6).

For all these characteristics, Pilates is useful for studying the two types of perspectives of imagination. To this aim, we have chosen two classical Pilates exercises of different difficulty: shoulder bridge (SB) and standing roll down (SRD) (Figure 1).

Although both exercises require concentration, breathing, balance, control, precision, and fluidity of movement during execution, SRD is considered more difficult than SB [38]. In particular, SRD is performed standing and starts by flexing the neck down followed by rolling down of the spine, vertebra by vertebra, preventing flexion of the trunk forward, and back to the initial position rolling out the column gradually. Instead, SB is performed in the supine position with the knees bent and arms along the body, starting with the retroversion of the pelvis and its upwards climb, vertebra by vertebra, and return to the starting position (Figure 1). Moreover, SRD necessitates good flexibility by the performer, and therefore, a physical impediment, such as visceral fat, can mechanically limit the movements.

### 2.3. Experimental Procedure

After the demonstration offered by the instructor, the experimental procedure consisted of 3 phases (VMI-I, VMI-E, and execution) repeated for both Pilates exercises (SB and SRD) in all the 3 groups of participants. The imagination phases (VMI-I and VMI-E) were performed in random order in the same day and separated by at least thirty minutes. However, the execution phase was performed 3 days after the imagination phases, so as not to create influences between imagination and execution. The entire experiment was completed in two consecutive weeks. The procedure is summarized in Table 1, while Figure 1 shows an example of the two different exercises.

In the demonstration, the instructor showed the randomly chosen exercise (SRD or SB) individually to each participant. Subsequently (first and second phases), the participants had to image in VMI-I or in VMI-E the exercise that had been previously shown by the instructor. The order of the two modalities of imagination was randomized within subjects. In both conditions, the participants were asked to close their eyes from the beginning to the end of the imagined exercise to guarantee the highest possible correspondence to physical exercise.

Finally, after 3 days, in the execution phase (third phase), the participants performed the exercise.

The following week, all participants repeated the entire procedure for the other exercise.

All participants did a single repetition in order not to create further learning of the exercise during the imagination phase.

The times of imagination (in seconds) were compared with those of execution.

### 2.4. Chronometer Features

Imagination and execution times were measured using a commercial digital stopwatch (Samsung Galaxy S4). The participants received instructions for the use of the stopwatch before the imagination task, in which they had to start and stop the clock on their own. In particular, they pointed the finger on the display, closed the eyes, said “go,” and, at the same time, activated the stopwatch. To control that the registration was done correctly, even the experimenter recorded the times. In the execution task, the stopwatch was used only by the experimenter.

The times recorded have been measured in seconds.

### 2.5. Statistical Analysis

The recorded data were first tested for normality (Shapiro–Wilk's test) and homoscedasticity (Levene's test) and then analysed by a three-way mixed-model analysis of variance (ANOVA): group (Expert, Novice, and no-Practice) × condition (VMI-I perspective, VMI-E perspective, and execution) × exercise (SRD, SB); post hoc analysis for multiple comparisons (Duncan's test) have been calculated when appropriate. Differences were considered significant at the *P* < 0.05 level. Time in seconds has been used as a dependent variable.

## 3. Results and Discussion

**Figure 2:**
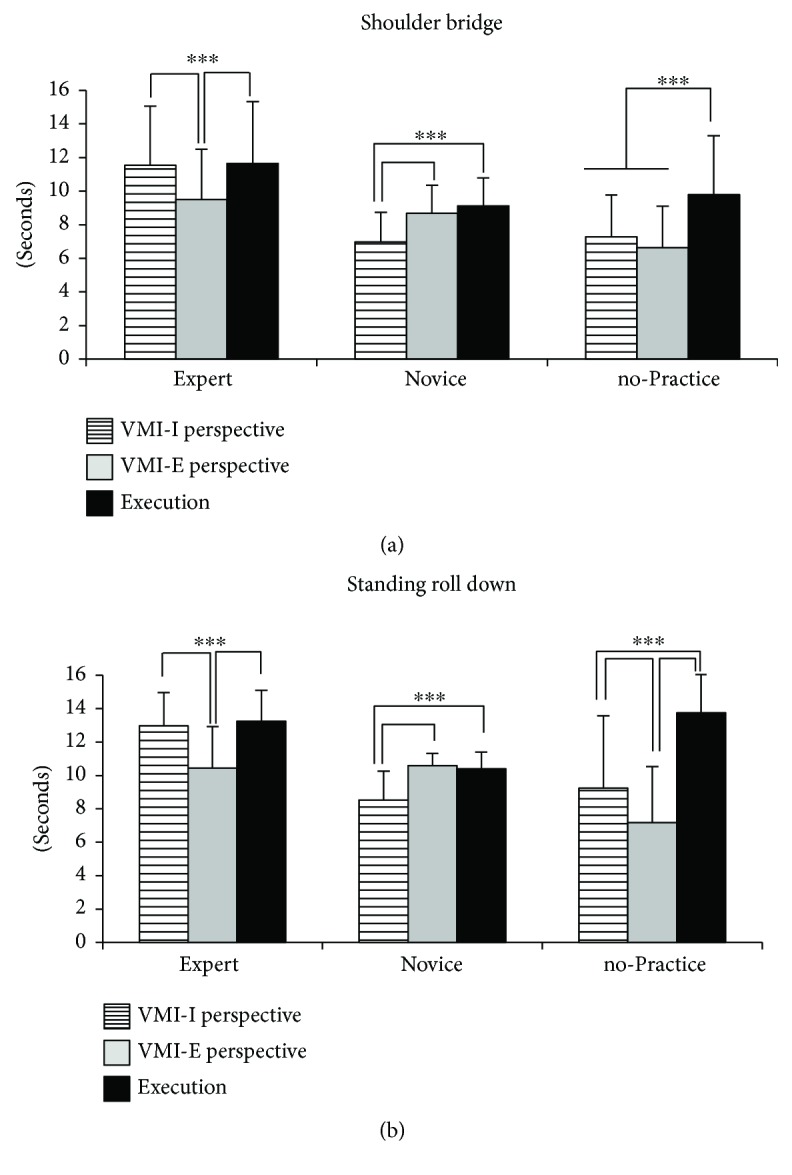
Execution and imagination times of the three experimental groups in both Pilates exercises: (a) VMI-I, VMI-E, and execution times in the shoulder bridge exercise; (b) times of both imagination perspectives and execution in the standing roll down exercise. Data are expressed as average ± SD. The asterisks indicate the significance level of the post hoc comparisons among groups (^∗∗∗^*P* < 0.0001).

As shown in Figures 2(a) and 2(b) and in Table 2, in the VMI-I, the Expert group needed the same time to imagine and perform both the Pilates exercises, while in the VMI-E, it took them less time to imagine than to perform both exercises. An opposite pattern was found in the Novice group, in which a significant time difference was present between VMI-I imagination and execution, in both exercises. In particular, it took them less time to imagine than to perform both exercises in VMI-I, while the same time was needed for imagination and for the execution in VMI-E. In the no-Practice group, both modalities of imagination for each exercise required a significantly lower time than the execution.

The present data evidenced that each modality of imagination is related to a different level of motor experience.

More specifically, the ANOVA revealed a statistically significant main effect for group (*F*_(2, 45)_ = 5.08; *P* = 0.01). Post hoc comparisons showed that the Expert group differed significantly from the other two groups, while the Novice and no-Practice groups were similar (Expert versus Novice: *P* = 0.009; Expert versus no-Practice: *P* = 0.01; Novice versus no-Practice: *P* = 0.94). Also, the condition factor showed a significant main effect (*F*_(2, 90)_ = 54.94; *P* = 0.00001). Post hoc comparisons showed that each condition was significantly different from the others (VMI-I versus VMI-E perspective: *P* = 0.02; VMI-I perspective versus execution: *P* = 0.01; VMI-E perspective versus execution: *P* = 0.0001). Finally, the types of exercise were statistically different (*F*_(1, 45)_ = 25.71; *P* = 0.00001), with SRD requiring more time (10.71 ± 2.12) than SB (9.02 ± 0.83). The second-order interaction was also significant (*F*_(4, 90)_ = 5.68; *P* = 0.0004), and the relative post hoc comparisons are shown in Figure 2. Furthermore, the interactions group × condition (*F*_(4, 90)_ = 23.81, *P* = 0.00001) and exercise × condition (*F*_(2, 90)_ = 5.19, *P* = 0.007) resulted to be statistically significant.

From these results, different conclusions can be drawn, in line with our hypothesis. In fact, we asked ourselves whether the two types of visuomotor imagery could have a different role in promoting learning and could have different applications in sport.

The similar times of VMI-I and execution and the dissimilar times of VMI-E and execution in experts (Figure 2 and Table 2) suggest that the level of motor competence induces a mental representation of the gesture. Such evidence is in line with Jeannerod [15] who underlined that to successfully employ VMI-I, it is necessary to have well-developed motor representations. It was then proposed that VMI-I might better suited for high-competence athletes [32]. Furthermore, VMI-I could be considered as a sort of “readiness for action” [39]. To this regard, it has been reported that VMI-I produces an increased electrical activity in muscles involved in the imagined activity with respect to VMI-E [40]. Conversely, the similar times of VMI-E and execution and the dissimilar times of VMI-I perspective and execution in the Novice group (Figure 2 and Table 2) suggest that VMI-E is facilitated by the allocentric reference frame. Probably, the novice, having to improve the gesture, uses the VMI-E perspective mainly to promote motor learning and to acquire behavioural skills. According to our interpretation, the VMI-E perspective represents a mental way to improve the execution of a sequence of movements [41] through a sort of “mental observation” of the background of the scene that induces skill acquisition.

Functional studies have correlated the brain activation to the competence levels. Olsson and coworkers [32] have evidenced that experts activate mainly motor areas and the cerebellum during the imagination of a sequence of movements (as long as such sequence belonged to their motor repertoire), while novices mainly activated the visual areas, thus suggesting functional differences in mental imagination related to experience. Cerebellar activation during imagery supports the internal model theory [42]. According to this concept, the cerebellum forms (through a learning process) an internal model that reproduces the dynamics of a body part [43]. In this line, VMI-I could be related to an internal model that allows neural circuits to precisely put the movement into action, without the need to refer the feedback from the moving body part [43].

The interpretation about a functional role of the two imagination perspectives is supported also by other functional imaging, neuropsychology, and lesion data that show that different cerebral areas are activated during these two modalities of imagination [21]. In particular, medial cortical structures (comprising anterior medial prefrontal, medial parietal, and posterior cingulate cortices) and the inferior lateral parietal cortex have been identified as the basic neural mechanisms involved in VMI-I, while the superior parietal lobe bilaterally (predominantly on the right side) and the right premotor cortex are associated with VMI-E [21].

The significant difference between both imagination conditions and execution times in the no-Practice group (Figure 2 and Table 2) suggests that imagery is not particularly helpful in the first phases of motor learning. As shown in Figure 2, regardless of the difficulty of the exercises, in both perspectives, the no-Practice group imagines the sequences of movements much faster than the actual execution, suggesting a reduced and limited knowledge and awareness of the exercise itself.

Another point to consider is the difficulty to imagine such exercises. As specified in Materials and Methods, SRD is more difficult than SB. Despite this difference, experts and novices obtained similar imagination and execution times even in the more difficult exercise in using the VMI-I for experts and the VMI-E for novices. Instead, for the SRD Pilates exercise, the no-Practice group obtained lower time imagination in VMI-E as compared to VMI-I probably since as the difficulty increases, the VMI-E imagination becomes faster because some movement or environment elements are omitted.

As a potential limitation of the study, we have to highlight that the entire sample was composed exclusively by women. This choice was not wanted but dictated by recruitment availability, since women practice Pilates more than men [44]. Although gender differences in motor imagery are not found frequently [45], some studies reported that females use less VMI-I perspective and more VMI-E perspective than males [19].

Another limitation of the study could be the fact that in the present paradigm, the subjects performed a single trial of each condition. Although we retain that this is a way to prevent the learning of the exercise during the imagination phases, further studies will be necessary to investigate VMI perspectives with more repetitions of the exercises.

Our experiment evidenced that the type of imagery is correlated to motor experience and independent from the type of sport or discipline, open or closed skill. Further studies are needed to clarify whether gender differences in the VMI perspectives really exist and what is their effective magnitude.

## 4. Conclusions

The present study allows us to draw different conclusions.

Firstly, visuomotor imagery is not a unique process. In fact, we propose that the internal view perspective serves to train or to revise a gesture already internalised. However, to reach such scope, a previous high motor competence level is needed. Indeed, the external view perspective, characterizing novices' abilities, serves to improve the learning of the gesture throughout “mental observation.” Finally, the visuomotor imagery in this context is not useful for individuals that lack the specific motor experience.

Although the present preliminary data offer insights on the application of mental training techniques, further studies are needed to analyse this topic in males.

## Figures and Tables

**Table 1:** Experimental procedure. Each participant started randomly with either shoulder bridge or standing roll down.

Demonstration	Pause	Phase 1	Pause	Phase 2	Interval	Phase 3
*Shoulder bridge (SB)*						
The instructor shows the SB exercise.	30 min	VMI-I perspective: imagine performing SB with closed eyes and with the body in the starting position (supine position)	30 min	VMI-E perspective with closed eyes: imagine the instructor performing SB starting from the initial position (supine position)	3 days	Execution: all participants performed the SB exercise
*Standing roll down (SRD)*						
The instructor shows the SRD exercise.	30 min	VMI-I perspective: imagine performing SRD with closed eyes and with the body in the starting position (standing position)	30 min	VMI-E perspective with closed eyes: imagine the instructor performing SRD starting from the initial position (standing position)	3 days	Execution: all participants performed the SRD exercise

**Table 2:** Mean time and standard deviation of the three experimental groups in the shoulder bridge (SB) and the standing roll down (SRD) Pilates exercises.

Group	SB			SRD		
	VMI-I	VMI-E	Execution	VMI-I	VMI-E	Execution
Expert	11.54 ± 3.52	9.50 ± 2.99	11.65 ± 3.67	12.99 ± 1.97	10.44 ± 2.49	13.25 ± 1.84
Novice	6.98 ± 1.75	8.68 ± 1.67	9.12 ± 1.67	8.53 ± 1.74	10.59 ± 0.73	10.40 ± 1.01
no-Practice	7.29 ± 2.49	6.63 ± 2.47	9.79 ± 3.52	9.24 ± 4.33	7.18 ± 3.35	13.76 ± 7.59
